# Changes of crystalline structure and physicochemical properties of *Pueraria lobata* var. *thomsonii* starch under water deficit

**DOI:** 10.1371/journal.pone.0304373

**Published:** 2024-07-03

**Authors:** Dan Gao, Xin Li, Fengyu Li, Rui Luo, Haimin Liao, Jianmin Man

**Affiliations:** Key laboratory of Plant Resource Conservation and Germplasm Innovation in Mountainous Region (Ministry of Education), Collaborative Innovation Center for Mountain Ecology & Agro-Bioengineering (CICMEAB), College of Life Sciences/Institute of Agro-bioengineering, Guizhou University, Guiyang, China; Universidad Tecnica de Ambato, ECUADOR

## Abstract

Crystal type is an important physicochemical property of starch. However, it is currently unclear whether changes in crystal type affect other properties of starch. This study discovered that water deficit resulted in an increase in small starch granules and transparency in *Pueraria lobata* var. *thomsonii*, while causing a decrease in amylose content and swelling power. Additionally, the crystal type of *P*. *Thomsonii* starch changed from C_B_-type to C_A_-type under water deficit, without significantly altering the short-range ordered structure and chain length distribution of starch. This transformation in crystal type led to peak splitting in the DSC heat flow curve of starch, alterations in gelatinization behavior, and an increase in resistant starch content. These changes in crystalline structure and physicochemical properties of starch granules are considered as adaptive strategies employed by *P*. *Thomsonii* to cope with water deficit.

## Instruction

*Pueraria lobata* var. *thomsonii* is a perennial deciduous vine belonging to the genus *Pueraria lobata*, which starch content of tuberous root can reach over 60% [1]. The physicochemical properties of starch are important factors affecting the starchy food products quality, which are not only determined by their genetic characteristics but also influenced by environmental factors. Water is a crucial environmental factor that significantly affects starch accumulation in tuberous root and starch properties. Starch accumulation is associated with both its synthesis and degradation processes. Under water deficit conditions, two scenarios may occur: a decrease in starch synthesis [2] or a decrease in osmotic potential due to sugars originating from starch degradation [3]. Depending on the balance between carbohydrate synthesis and consumption, soluble sugars and starch can be transformed into one another at various stages of plant life [4].

The regulation of starch synthesis and breakdown in plants under water deficit can significantly alter starch structural properties, especially the type of crystalline structure, which can be divided into A-, B-, and C-type by X-ray diffraction pattern [5]. The A-type crystalline structure is present in cereal starches such as wheat, rice and maize starches. During the grain filling period in wheat, water deficit can inhibit starch accumulation, alter starch size distribution, and reduce amylose, amylopectin, and total starch content. But the crystal type (A-type) of endosperm starch remains unchanged [6]. Zhang et al. [7] investigated the changes in rice starch fine structures and functional properties induced by water deficit and elevated CO_2_, and under the water deficit condition, the crystal type of starch granules did not change and remained A-type. Water deficit also had not changed on the crystalline allomorphs of the normal maize starch [8]. Zhang et al. [9] obtained the same conclusion in the study of water deficit wheat: A-type crystalline structure maybe stable under water deficit. However, the effects of water deficit on B- and C-type starches are less reported.

The C-type starch contains both A- and B-type crystallinity [10]. Genkina et al. [11] showed that sweet potato tubers which contain C-type starch were more likely to form and accumulate A-type crystallinity when the soil temperature was above 33°C, whereas C-type crystallinity was more likely to form and accumulate in sweet potato tubers when the soil temperature was below 15°C. A-type crystallinity may be more resistant to adverse conditions. Almeida et al. [12] found that crystal type of *Trimezia juncifolia* starches differed in the dry and wet seasons, with the A-type in the dry season and C-type in the wet season, and the crystallinity degree in the wet season was higher than in the dry season, which was correlated to the different moisture conditions. In summary, the accumulation of A-type crystallinity in plants is an adaptive response to adverse conditions because the A-type crystallinity is more perfect than B-type crystallinity. Changes in the starch crystallinity may be caused by changes in the activities of starch synthase (SS), starch branching enzyme (SBE), and debranching enzyme (DBE), as the crystalline regions are mainly composed of amylopectin synthesized through above enzymes [13]. Crystal distribution C-type starch is more complex than A- and B-type starch, which leads to the special gelatinization properties of C-type starch. Coexistence of A-, B-, and C-type starch granules has been observed in sweet potato root tubers [14], while C-type starches from pea and Chinese yam have been found to have A- and B-type crystals on both the outside and inside of the same granules [15–17]. Starches with different crystal types exhibit varying gelatinization characteristics. A-type and B-type starches have typical unimodal DSC curves with a narrow gelatinization temperature range. On the other hand, C-type starches have a broad DSC endothermic peak and may even display two or three endothermic peaks [18]. It remains uncertain whether environmental factors can induce changes in the crystal type of *P*. *thomsonii* starch, a typical C-type starchy plant, and if such changes are associated with alterations in pasting properties.

*P*. *Thomsonii* is a plant species widely distributed in the southwest China. It thrives in warm, moist, well-drained, sandy soils with good drainage. However, the region is characterized by its mountainous terrain, which in some areas have severe rocky desertification, unfavorable irrigation, and poor soil water retention capacity. Due to its susceptibility to water deficit, *P*. *Thomsonii* experiences decreased energy and yield. Currently, there is limited research on *P*. *Thomsonii* response to water deficit. It has been observed that water deficit considerably impedes the expansion of root tubers [19]. However, there is a limited amount of research on the regulation of kudzu starch crystal assembly under water deficit. Investigating the impact of water deficit on different types of starch crystals, as well as their physical and chemical properties, can provide valuable insights into the mechanism of starch synthesis and assembly in organisms.

## Materials and methods

### Plant material and treatment conditions

*P*. *Thomsonii* seedlings (purchased from Teng County, Guangxi Province, China) with approximate growth status were selected for water deficit. The potting soil used in the experiment was a mixture of surface soil of farmland and humus soil in a volume ratio of 3:1, with soil moisture content maintained at field capacity (55%). To ensure soil moisture content remained stable throughout the experimental period, a combination of natural water consumption and artificial water replenishment was used. The water deficit treatment phase was initiated on September 28th, 2020. Three moisture content gradients were established: the relative soil moisture content (ratio of soil moisture content to field water capacity) of the control treatment (CK) was 70–80%, and the relative soil moisture content of the two water deficit treatments was maintained at 40–50% (D1) and 25–35% (D2). After 4 weeks of water deficit treatment, root tubers were took out, cleaned and then stored in ziplock bags at -20 °C for use.

### Starch extraction

Frozen root tubers were thawed and cut into slices. The pieces were homogenized in fresh water using a home blender. The homogenate was then pressed by hand through four layers of gauze. The extract was filtered with 100-, 200-, and 400- mesh sieves and centrifuged at 5000 rpm for 10 min. After each centrifugation, a supernatant was discarded and a yellow-brown material was scraped off from the top layer of precipitated starch. This was repeated until the surface of the precipitate was white. Finally, the samples were dried at 40 °C, ground into powders, and passed through a 100-mesh sieve.

### Starch content determination

The content of starch was measured following the method described in the reference [20] with some modifications. Briefly, frozen root tubers were thawed and sliced, dried at 45 °C to constant weight, ground to powder in a grinder and sieved through a 100-mesh sieve. 100 mg of powder was mixed with 2 mL of distilled water and 3.2 mL of 60% (v/v) perchloric acid, stirred for 10 minutes, the solution final volume was made up to 100 mL with distilled water. 0.5 mL of the starch solution was taken, 3 mL of distilled water and 2 mL of iodine solution (0.2% I_2_, 2% KI, w/v) were added, allowed to stand for 5 min and measure the absorbance at 660 nm.

### Scanning Electron Microscope (SEM) observation

A certain amount of starch powder was applied to an aluminum stub using double-sided adhesive tape, coated with gold using a sputter coater. The starch samples were observed and photographed in a SEM (S-3400N, Hitachi Ltd, Tokyo) at 1200 × magnifications with accelerating voltage of 20 kV.

### Granule size analysis of starch

A laser particle size analyzer (Bettersize 2600, Dandong Better Instruments, China) was used to measure the particle size of *P*. *Thomsonii* starch. The refractive index of the sample and dispersant were set to 1.33 and 1.60, respectively, and the shading degree was set to 5–8%.

### Amylose content determination

The amylose content was determined based on the method described in the reference [21] with some modifications. 10 mg of *P*. *Thomsonii* starch was suspended in 5 mL of 90% (v/v) aqueous dimethyl sulfoxide (DMSO). The mixture was incubated at 95 °C for 30 minutes to obtain a clarified solution, which was then cooled to room temperature. 1 mL of starch-DMSO solution was immediately mixed with 1 mL of iodine solution (0.2% I_2_, 2% KI, w/v), and then adjusted the final volume to 50 mL with distilled water. The solution was mixed well andplaced for 30 minutes in the dark. Amylose content was calculated from absorbance at 620 nm and referring to the standard curve, which was prepared using amylopectin from corn (Sigma, USA) and amylose from potato (Sigma, USA).

### Amylopectin chain length distribution

*P*. *Thomsonii* starch was debranched using isoamylase (Sigma, USA) by the method described in the reference [22]. The chain length distribution of branched starch was analyzed by high-performance anion-exchange chromatography (HPAEC) with pulsed amperometric detection (PAD; ICS500+, Thermo Fisher Scientific, USA). Technical support for HPAC-PAD and amylopectin chain length analysis was provided by Sanshu Biotech. Co. Ltd (Shanghai, China).

### X-ray diffraction (XRD) analysis

XRD analysis was performed on an X-ray diffractometer (Empyrean, PANalytical B.V., Netherlands) [23]. All samples were stored in a closed container with sodium chloride saturated solution to maintain a constant humidity to absorb water for 1 week before testing. The test conditions were as follows: 40 kV, 40 mA, 2θ scanning range from 5° to 40°, scanning speed 1.2 °/min, and step length 0.02°. The method described by Huang et al. [24] was used to calculate the relative crystallinity of starch.

### Attenuated total reflectance-flourier transform infrared (ATR-FTIR) analysis

An ATR-FTIR (Nicolet iS50, Thermo Fisher Scientific, USA) spectrometer was used to analysis short-range ordered structures of starch [25]. The scanning range was 4000–800 cm^-1^, and OMNIC software was used for deconvolution. The assumed line shape was Lorentzian with a half-width of 19 cm^-1^ and a resolution enhancement factor of 1.9. The intensity values at 1047, 1022 and 995 cm^-1^ were extracted from the spectra after water subtraction, baseline correction and deconvolution. The intensity ratio of 1045/1022 cm^-1^ and 1022/995 cm^-1^ was calculated.

### Solubility (SOL) and swelling power (SP) determination

The SOL and SP were determined based on the method described in the reference [26] with some modifications. A 30 mg starch sample in a pre-weighed centrifuge tube (*m1*) was suspended with 2 mL of distilled water and mixed using vortex. The mixture was heated at 95°C for 30 minutes, cooled to room temperature and centrifuged at 8000 rpm for 10 minutes. Carefully transferred the supernatant to another pre-weighed centrifuge tube (*m2*), and the sediment with centrifuge tube was weighed (*m3*). The centrifuge tube with supernatant was baked at 105 °C for 24 h until constant weight, and weighed (*m4*). The SOL and SP of starch were calculated as follows:

$$\text{SOL}\left(\%\right)\;=\;\frac{\text{m}4-\text{m}2}{\text{m}}\times 100\%$$ (1)

$$\text{SP}\left(\text{g}/\text{g}\right)\;=\;\frac{\text{m}3-\text{m}1}{\text{m}\times \left(1-\text{SOL}\%\right)}$$ (2)

### Transparency determination

The determination of transparency based on the method described in the reference [27]. Accurately weighed 50 mg of starch into a 10 mL plastic centrifuge tube, added 5 mL of distilled water and mixed to make 1% (w/v) starch milk. It was heated into a paste, treated at 95 °C for 30 minutes, and then cooled to room temperature. The light transmittance (%) was measured at a wavelength of 650 nm as the transparency, with distilled water setting as a blank.

### Differential scanning calorimetry (DSC) analysis

The thermal properties of samples were determined using differential scanning calorimetry (Q2000, DSC, TA Instruments, USA) [28]. 3 mg of starches and 9 μL of ultra-pure water were placed into an aluminum crucible, placed at 4 °C for 24 hours. After equilibrating at room temperature for 1 h, the crucible was heated from 25 °C to 130 °C at 10 °C/min with an empty crucible as reference. Peak fitting was performed using PeakFit v4.12.

### Observation of gelatinization behavior of starch

A 20 μL starch suspension (3%, w/v) was applied to a glass microscope slide. The slide was then covered with a glass coverslip and sealed with nail polish to prevent moisture loss during heating. The sealed glass slide was placed in the center of the heating stage and heated from 25 °C to 50 °C at a rate of 5 °C/min. Subsequently, it was heated from 50 °C to 70 °C at a rate of 2 °C/min. After removing the slide, it was immediately observed under a polarizing microscope (Axio Scope AI A, Carl Zeiss, Germany) and pictures were taken for recording. The degree of gelatinization (DG) of starch at a specific temperature was determined by calculating the percentage of gelatinized granules to the total number of starch granules in the visual field. Starch granules that lacked birefringence were considered to be gelatinized starch [14].

### In-vitro digestion of starches

The content of rapidly digestible starch (RDS), slowly digestible starch (SDS), and resistant starch (RS) in the samples was analyzed [29]. Weighed 0.3 mg of porcine pancreatic α-amylase (Sigma, USA) into 4 mL of 0.02 mol/L (pH 6.9) sodium phosphate buffer, and vortex mixed for 20 minutes. The supernatant collected by centrifuge (8,000 rpm, 10 min) was the enzyme solution. 10 mg starch glucosidase (Sigma, USA) was shaken with 1 mL 0.02 mol/L (pH 4.8) acetic acid buffer in a centrifugation tube and then transfer the above two enzyme solutions to a new centrifuge tube and mixed well. 100 mg of starch, 4 mL of 0.5 mol/L sodium acetate buffer (pH 5.2), 1 mL of enzyme solution were shaken well with 5 glass beads in a 10 ml centrifuge tube which was incubated in a shaking water bath (37 °C, 200 r/min). Took out the centrifuge tube at 20 min and 120 min respectively, and centrifuged at 8500 rpm for 5 min. The supernatant (0.1 mL) was taken and mixed with 1 mL of 80% ethanol. Then, 1 ml of 3, 5-dinitrosalicylic acid (DNS) was added and heated at 95 °C for 5 min to measure the starch degradation rate at 20 min and 120 min, which are recorded as G20 and G120. Determined values of different concentrations of glucose standard solutions were used to construct the standard curve. RDS, SDS and RS were calculated as follows:

$$\text{RDS}\left(\%\right)\;=\;\text{G}20\left(\%\right)$$ (3)

$$\text{SDS}\left(\%\right)\;=\;\text{G}120\left(\%\right)-\text{G}20\left(\%\right)$$ (4)

$$\text{RS}\left(\%\right)\;=\;100\%-\text{RDS}\left(\%\right)-\text{SDS}\left(\%\right)$$ (5)

### Statistical analysis

The SPSS 26.0 Statistical Software Program were used for analysis of variance (ANOVA) by Tukey’s test (*p* < 0.05) and Origin 2018 was used for plotting.

## Results and discussion

### Chemical composition

The total starch content (the mass fraction of starch) in D1 was not significantly different from that of the control group (CK), while the total starch content in D2 was significantly lower than that of CK (Table 1), which was consistent with the study of Wang et al. [30] that water deficit reduced total starch content in cotton. In our previous research [19], it was observed that water deficit in *P*. *Thomsonii* can inhibit leaf expansion, which in turn restrict photosynthesis and hinder root expansion, this may suggest that the total amount of starch in *P*. *Thomsonii* root decreased. These results suggest that although the total amounts of starch reduced under a certain degree of water deficit (D1), the degree of starch filling did not change with the obstruction of root enlargement, unless there is more severe water deficit (D2). There was no significant difference (*p*<0.05) in the amylose content of *P*. *Thomsonii* starch under water deficit.

**Table 1:** Effects of water deficit on starch granule size distribution of starches.

Sample	Total starch content (%)	Amylose content (%)	Median particle size	Small starch granules	Middle starch granules	Large starch granules
			(μm)	≤5 μm (%)	5–15 μm (%)	≥15 μm (%)
CK	23.12±2.123a	25.25±0.019a	8.011±0.002a	23.387±0.038c	44.260±0.000a	32.353±0.038a
D1	24.51±1.259a	24.71±0.008a	7.013±0.022b	35.190±0.165b	39.953±0.090b	24.857±0.255b
D2	20.34±1.065b	23.94±0.014a	6.048±0.028c	44.373±0.270a	36.407±0.075c	19.220±0.345c

Mean ± SD is calculated from duplicate measurements.

Values with different letters in the same column are significantly different (*p*< 0.05).

### Starch granule morphology characteristics

**Fig 1:**
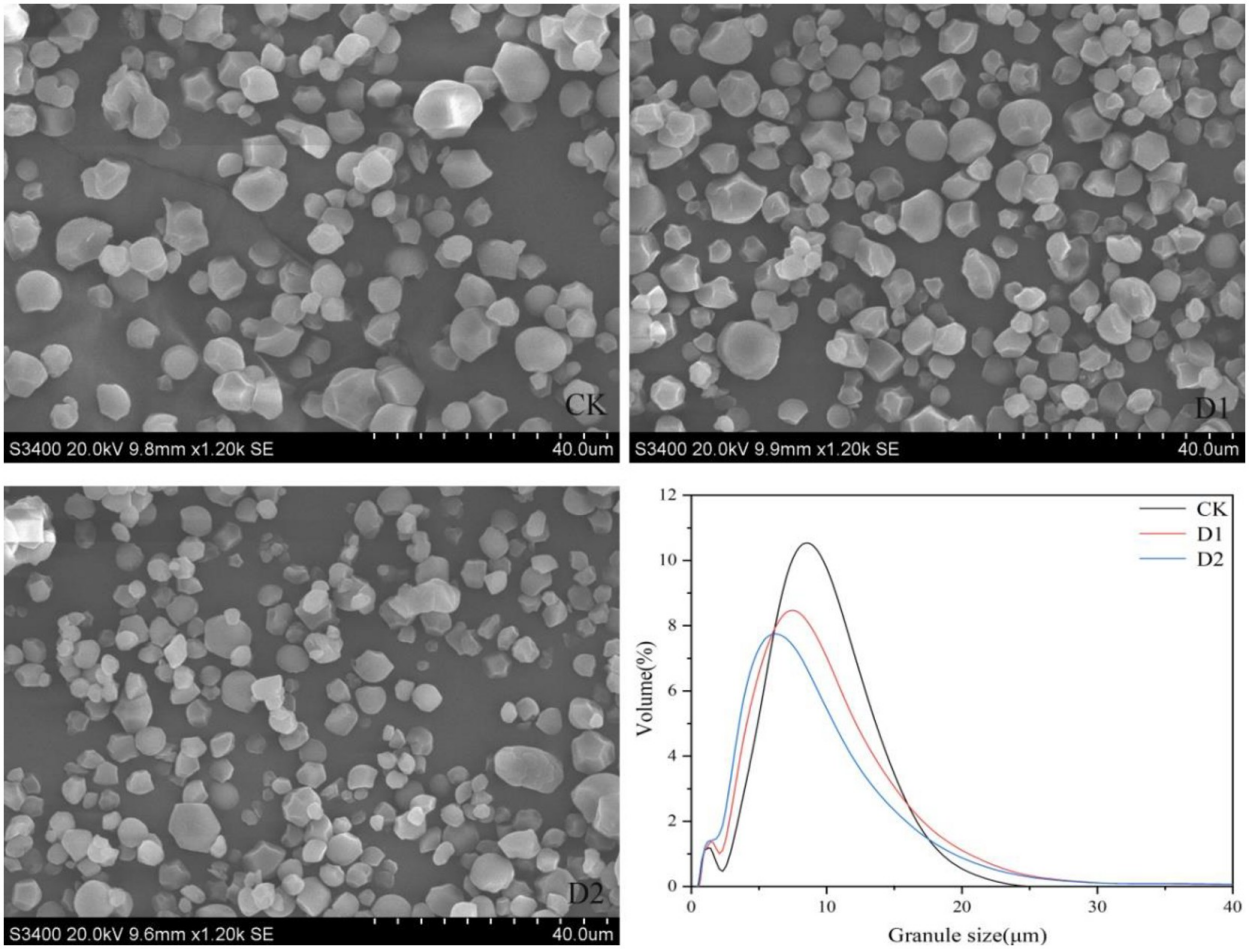
Morphology, and particle size distribution of starches. CK, soil relative water content was 75%±5%; D1, soil relative water content was 45%±5%; D2, soil relative water content was 30%±5%.

Starch granule morphology varies depending on the plant sources. Genetic factors play a significant role in determining the shape and size of starch granules, but environmental factors play equally important roles. Moisture, temperature, and photoperiod are some of the environmental factors that can interfere with the morphological characterization of starch granules. Among these factors, moisture has the most significant impact on particle size [31]. This work examined the shape and size of starch granules in *P*. *Thomsonii* under water deficit. The shape of the granules did not have a significant response to water deficit, mainly polygonal. However, SEM pictures (Fig 1-D1 and 1-D2) revealed an increase in the number of small starch granules under water deficit. The particle size distribution is a critical parameter in determining starch structural characteristics. Previous studies have categorized starch into three types based on granule size: large (≥15 μm), medium (5–15 μm), and small (≤5 μm) starch granule [32]. The results of particle size distribution analysis showed a bimodal distribution, with an increase in small starch granules and a decrease in large and medium granules under water deficit (Fig 1, Table 1). These findings suggest that water deficit significantly affects the proportion of different types of starch granules in *P*. *Thomsonii*. Median particle size is an important index to measure the grain size distribution of starch grains, and water deficit significantly reduced the median particle size of *P*. *Thomsonii* starch grains (Table 1). The amylose content is higher in large starch granules than in small ones, and this may be related to granule bound starch synthase (GBSS) [33]. Water deficit reduced GBSS activity, resulting in decreased amylose synthesis and increased synthesis of small starch granules while decreasing the synthesis of large starch granules. Zhang et al. [32] found that water deficit reduced the accumulation of small starch granules, but increased the content of large starch granules. At the same time, water deficit had a significant effect on the median size of wheat endosperm starch granules volume distribution, which indicated that the effects of water deficit on the grain size distribution of starch from different plant sources were different.

### Chain length distribution of amylopectin

**Fig 2:**
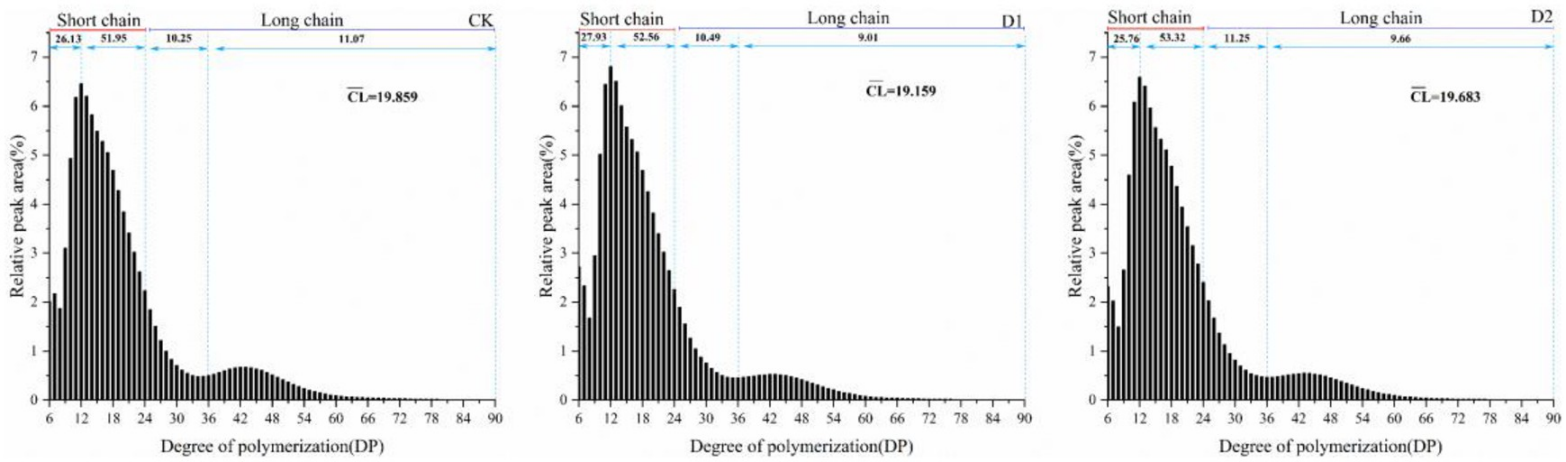
Amylopection chain length distribution of starches. CK, soil relative water content was 75%±5%; D1, soil relative water content was 45%±5%; D2, soil relative water content was 30%±5%.

According to the degree of polymerization (DP), the amylopectin side chains can be divided into A chains (DP 6–12), B1 chains (DP 13–24), B2 chains (DP 25–36) and B3 chains (DP≥ 37) [34]. In general, amylopectin chains can be divided into two main groups, short (DP≤24) and long chains (DP>24), with different arrangements depending on the starch source [35]. The short chains are typically anchored to the long chains, which act as linker chains, and are mainly found in the amorphous lamellar structure of starch granules [36]. The content of different amylopectin side chains can be expressed by the proportion of peak are as in different polymerization degree ranges in the chain length distribution spectrum. Chain length distributions of amylopectin revealed a decrease in the proportion of amylopectin long chains and average chain length ($\bar{\text{CL}}$) with a concomitant increase in the short chains in *P*. *Thomsonii* starch grains under water deficit (Fig 2). Under water deficit, the increase in short chains of amylopectin may be linked to the higher activity of branching enzymes that synthesize shorter chains of amylopectin. The ATP energy cost for the degradation of short chains is lower, making them preferentially utilized during water deficit [37].

### Crystalline structure of starches

**Fig 3:**
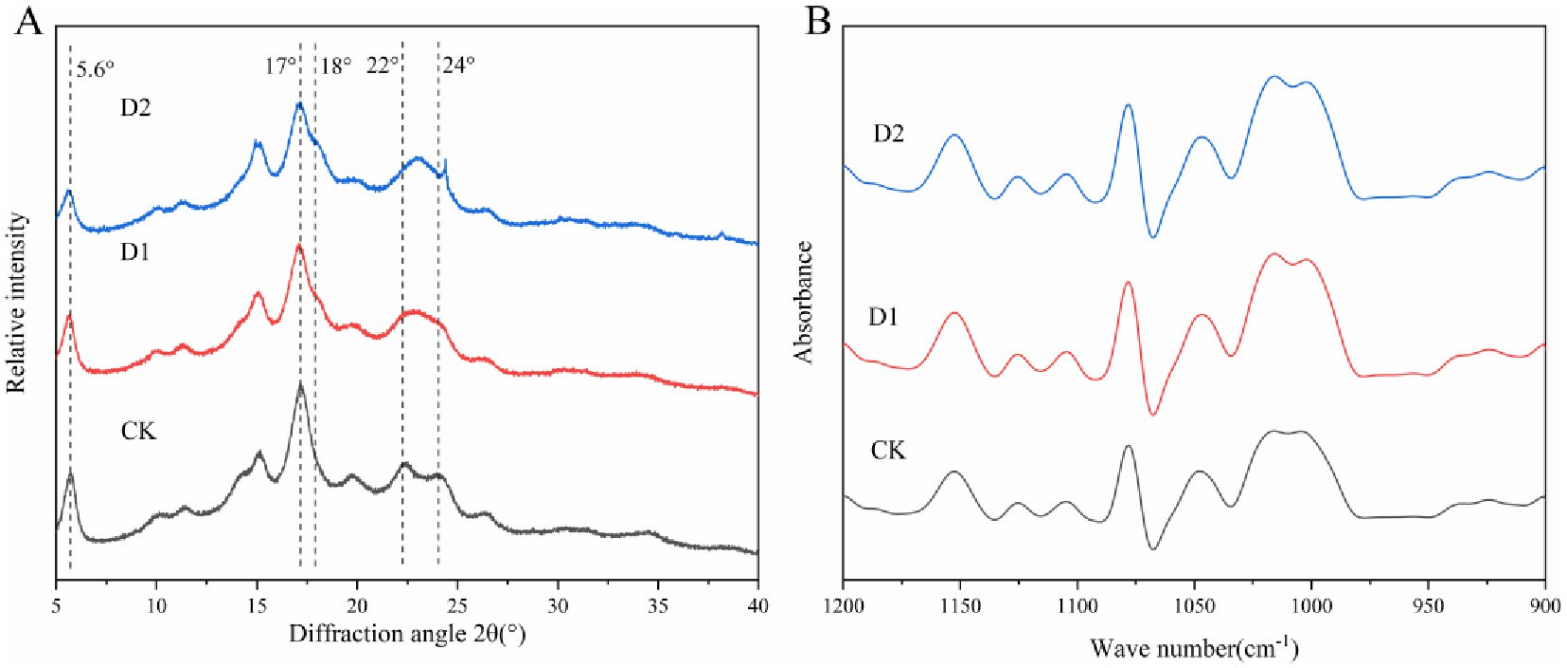
XRD (A) and FTIR (B) patterns of starches. CK, soil relative water content was 75%±5%; D1, soil relative water content was 45%±5%; D2, soil relative water content was 30%±5%.

XRD can be used to detect the crystalline structure and measure crystallinity of granule starch. Based on XRD patterns, crystal types of starches from different sources normally can be classified into three types: A-, B-, C-type. C-type starch is generally considered to be mixtures of A-type and B-type crystallinity [38]. According to the difference in XRD patterns caused by the proportion of A- and B-type crystallinity, C-type starch can be further divided into C_A_-, C_B_-, C_C_-type [10]. As portrayed in Fig 3A, the native *P*. *Thomsonii* starch had a strong diffraction peak near 2θ 17° and connected double peaks near 2θ 22° and 24°, in accord with a C_B_-type diffraction pattern. *P*. *Thomsonii* starches under D1 and D2 treatments showed typical C_A_-type diffraction pattern with two shoulder peaks at about 2θ 17° and 18°, a singlet at about 2θ 23° in D2 and obscure double peaks at 2θ 22 and 24° in D1. The results of this study showed that that subjecting *P*. *Thomsonii* to water deficit treatment resulted in a transformation of its starch crystallinity from C_B_-type to C_A_-type, which indicated water deficit could hinder the formation of B-type crystallinity. In A-type polymorph, double helices formed by amylopectin are packed within a monoclinic unit cell with 4 water molecules. On the other hand, in B-type polymorph, amylose and amylopectin are intertwined, and the double helices are packed in a hexagonal unit cell associated with 36 water molecules [39]. The length of the amylopectin chain is also a decisive factor for crystal type of starch granules. Shorter branched chains and closed branch points of amylopectin favor A-type crystallinity, while longer branched chains and farther branch points favor B-type polymorph [40]. Under water deficit, short chains in the chain length distribution of amylopectin increased, so it tended to synthesize A-type crystallinity. Furthermore, the degree of transformation increased with the severity of water deficit.

The relative crystallinity degree, an important parameter for characterizing starch properties, was observed to decrease with the increasing severity of water deficit, as shown in Table 2. The transformation of crystal type is one of the main strategies by which plants can adapt to limited water availability. A decrease in starch particle size, an increase in the proportion of short amylopectin chains [41, 42], and a decrease in amylose content [43] could explain a decrease in the crystallinity degree of starch reported in this study. The transformation of crystal type may also be a factor, as B-type crystallinity have higher relative crystallinity than A-type crystallinity. Under water deficit, the reduction in crystallinity degree could improve the utilization of amylose [44].

**Table 2:** Relative crystallinity and infrared ratio of starches.

Sample	Relative crystallinity (%)	1045/1022 cm^-1^	1022/995 cm^-1^
CK	30.442±0.001a	0.665±0.063a	1.124±0.079a
D1	30.230±0.003a	0.646±0.055a	1.045±0.082ab
D2	28.702±0.001b	0.702±0.061a	0.887±0.070b

Mean ± SD is calculated from duplicate measurements.

Values with different letters in the same column are significantly different (*p*< 0.05).

### Short-rang ordered structure of starches

Attenuated total reflectance-Fourier transform infrared (ATR-FTIR) spectroscopy is used to characterize the short-range ordered structure of starch which is sensitive to changes in starch conformation. The ratio of 1045/1022 cm^-1^ can quantify the relative content between the ordered and amorphous structures of starch and the ratio of 1022/995 cm^-1^ can be used as an index of the proportion of amorphous to ordered carbohydrate structure in starch [25]. The ATR-FTIR deconvolution spectra of *P*. *Thomsonii* starches under water deficit were similar (Fig 3B). The ratio of 1045/1022 cm^-1^ increased slightly, and the ratio of 1022/995 cm^-1^ decreased (Table 2), the results indicated that *P*. *Thomsonii* starches under water deficit treatment became more ordered. Wang et al. [45] have shown that the order degree of waxy maize starch increases under water deficit, which is consistent with the findings of this study. Water deficit had less effect on the double-helical order of starch.

### Solubility, swelling power and transparency

Change in starch granule size will affect other properties of starch. The solubility significantly increased under D1 treatment compared to CK, while swelling power and solubility decreased under D2 treatment. Small starch granules have high lipid content, and the combination of lipids and amylose impedes the combination of water molecules and starch [46]. This may explain the reduction of starch swelling power in this study. The interference of water deficit with the activity of GBSS, resulting in a decrease in amylose content. Transparency increased with increasing water deficit degree, which was opposite to the change pattern of amylose content (Table 3). Starch molecules with high amylose content have a larger flow radius, greater steric hindrance in the starch paste, difficult formation of parallel orientation between molecules, better molecular dispersion, and more reflection and scattering when light passes through [45]. This results in lower transparency of the starch paste, hence the decrease in amylose content and increase in transparency.

**Table 3:** Effects of water deficit on swelling power, solubility and transparency of starches.

sample	Swelling power	Solubility (%)	Transparency (%)
CK	25.96±0.305a	21.43%±0.005b	17.70±0.10b
D1	24.71±1.031a	23.77%±0.004a	18.43±0.75a
D2	22.48±1.297b	20.63%±0.015b	18.53±0.38a

Mean ± SD is calculated from duplicate measurements.

Values with different letters in the same column are significantly different (*p*< 0.05).

### Thermal properties

**Fig 4:**
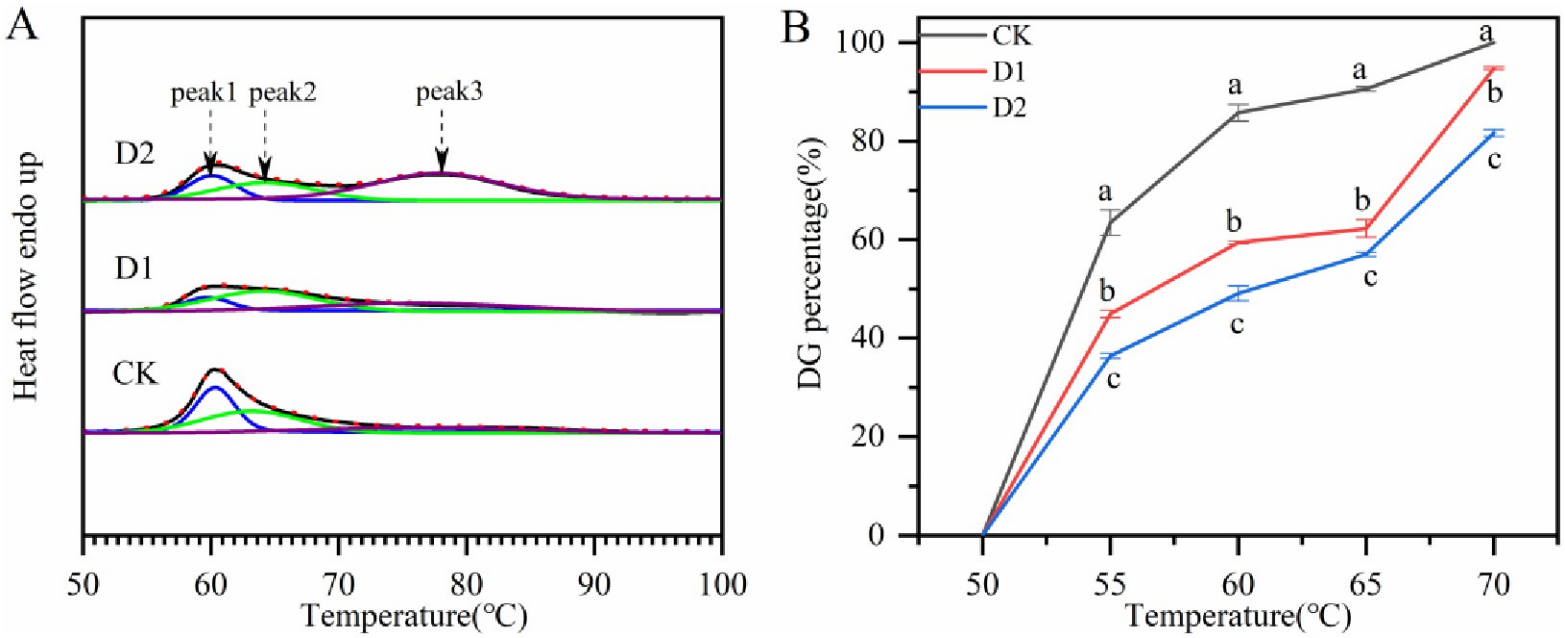
DSC thermograms and their peak-fitting profiles of starches (A) and the DGs of starch granules during heating process by hot stage polarizing microscope (B). The black curve is the original thermogram after baseline correction, the blue (peak1), green (peak2) and purple (peak3) curves are the fitted peaks, and the red dotted curve is the fitted thermogram (A). Vertical bars represent ± SD of the mean (n = 3), where they exceed the size of the symbol. Letters a, b and c represent statistical significance, p < 0.05 (B). CK, soil relative water content was 75%±5%; D1, soil relative water content was 45%±5%; D2, soil relative water content was 30%±5%.

Differential scanning calorimetry (DSC) is a common characterization method used to study the change of crystalline structure during starch gelatinization [47]. The DSC thermogram of native *P*. *Thomsonii* starch (CK) has shown a single endothermic peak. Starch in D1 treatment displayed inconspicuous double peaks and the peak width was obviously wider than that of CK in the DSC thermogram, while two separate peaks were observed in the DSC thermogram of starch in D2 treatment (Fig 4A). The wide DSC thermogram can be fitted into two or three peaks [14, 15]. The DSC thermograms were fitted with three peaks using PeakFit software, the fitting profiles and parameters are presented in Fig 4A and Table 4. The fitting results were basically consistent with the actual observation thermograms. According to the peak temperature from low to high, the fitting peaks were marked as peaks 1, 2, and 3, respectively. Generally, the gelatinization temperature of B-type crystallinity is lower than that of A-type [18], while the gelatinization temperature of C-type crystallinity is between A-type and B-type crystallinity [14]. Therefore, it was speculated that peaks 1 and 3 were the gelatinization peaks of B-type and A-type crystallinity, respectively, and peak 2 was the gelatinization peak of C-type crystallinity. Water deficit had no significant effect on the peak temperature of peak 1, but with the deepening of water deficit, the peak area decreased significantly. The peak temperature of peak 2 was significantly increased under water deficit, the area of peak 2 was significantly increased in D1, and the area of peak 2 was significantly decreased in D2. Under CK treatment, the area of peak 1 was larger than that of peak 3 (Table 4), indicating that the content of B-type crystallinity was higher than that of A-type, and the result was consistent with XRD analysis (C_B_-type crystallinity). The area of peak 1 was smaller than that of peak 3, indicating that the content of B-type crystals was less than that of A-type crystals, which was consistent with XRD analysis (C_A_-type crystallinity). This also indicated that water deficit caused the transformation of *P*. *Thomsonii* starch crystals, and the degree of transformation was related to the degree of water deficit.

**Table 4:** DSC parameters and the peak area percentage of peak-fitting profiles of starches.

Sample	peak1		peak2		peak3		r^2^
	Tp(°C)	Ap(%)	Tp(°C)	Ap(%)	Tp(°C)	Ap(%)	
CK	60.340±0.000a	36.812±0.465a	63.235±0.030b	40.444±0.400b	76.144±0.025b	22.717±0.086c	0.997
D1	59.766±0.012a	13.217±0.006c	64.172±0.098a	50.852±0.004a	75.968±0.002c	35.930±0.011b	0.997
D2	60.085±0.003a	17.845±0.370b	64.179±0.046a	27.605±0.286c	77.761±0.001a	54.549±0.085a	0.999

Mean ± SD is calculated from duplicate measurements.

Values with different letters in the same column are significantly different (*p*< 0.05).

Tp, peak temperature; Ap, peak area percentage; r^2^, the determination coefficient of peak fitting.

### Gelatinization behavior of starch granules and their DGs

**Fig 5:**
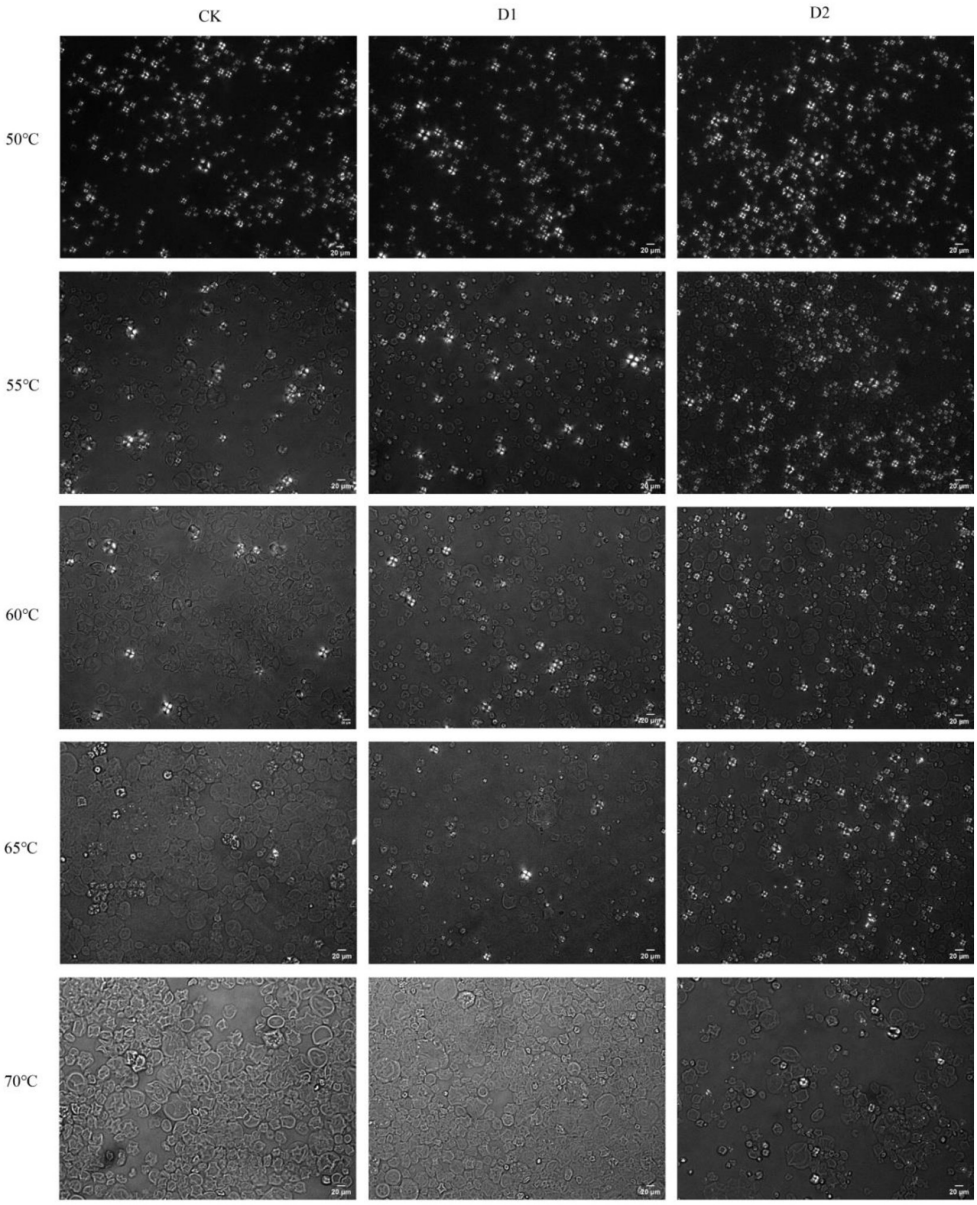
Gelatinization behavior of starches observed by hot stage polarizing microscope. CK, soil relative water content was 75%±5%; D1, soil relative water content was 45%±5%; D2, soil relative water content was 30%±5%.

The gelatinization behavior of starch granules can be observed using polarizing microscope with a hot stage. Starch granules are semi-crystalline and exhibit bright “Maltese crosses” under polarized light. Heating destroys the crystalline regions of starch granules, causing the “Maltese crosses” to disappear [48]. At 50°C, the starch exhibited intact granule morphology and birefringence crosses. Starch granules started to swell and lose birefringence crosses when heated to 55°C. There were clear differences in the starch gelatinization behaviors of CK, D1 and D2. Based on the gelatinization temperature of starch granules, they could be categorized into three groups [14]. The first group of granules underwent gelatinization below 60°C, the second group gelatinized from 60 to 65°C, and the third group gelatinized above 65°C. The granules in Group 1 and Group 3 corresponded to the first and third peaks, respectively, in the DSC fitting peaks. This indicates that Group 1 granules had a lower gelatinization temperature and were classified as A-type starch, while Group 3 granules had a higher gelatinization temperature and were classified as B-type starch. The granules in the second group corresponded to the second peak, which represented C-type starch (Fig 5). Similar results were also detected in starches from sweet potato [14]. As water deficit increased, the number of gelatinized starch granules above 65°C also increased, indicating an increase in A-type starch. The DGs of starch granules was measured during gelatinization. As the temperature increased, DGs of starch granules increased for all treatments at all temperatures. With the deepening of water deficit, the degree of gelatinization decreased at all temperatures (Fig 4B). All of the above indicated the water deficit treatment reduced the possibility of destroying the starch crystalline area.

### In vitro digestibility of starches

C-type starchy plants produce high levels of resistant starch (RS) and slowly digestible starch (SDS), which can decrease the in vivo hydrolysis rate of starch and are considered dietary fiber [49]. Compared with CK, both RDS content and RS content significantly increased under water deficit, whereas SDS content decreased significantly (Table 5). While previous studies have indicated that water deficit leads to an increase in RDS and SDS content, and a decrease in RS content, this study found that RS content increased under water deficit [50–52]. This could be due to several factors, including a decrease in the synthesis of B-type crystallinity and an increase in A-type crystallinity [53, 54], which is less susceptible to be attacked by α-amylase or glucoamylase [55]. Additionally, water deficit can lead to a reduction in starch particle size, which is directly proportional to digestibility. In other words, smaller starch particles are less digestible [56]. Finally, the proportion of ordered structure of starch increased under water deficit, but starch with a higher proportion of ordered structure was found to be less easily digestible [57]. Under water deficit, the content of SDS decreased, which was opposite to the proportion of A chains. The reason may be that the content of SDS was affected by the chain length of amylopectin, and the increase of the proportion of A chains would reduce the content of SDS [58, 59]. Crystalline type transformation may also be one of the reasons for the decrease in SDS.

**Table 5:** Effects of water deficit on in vitro digestion characteristics of starches.

Sample	RDS (%)	SDS (%)	RS (%)
CK	11.395±0.803b	13.476±0.281a	75.129±0.616c
D1	13.030±0.753a	5.077±0.242c	81.893±0.522a
D2	13.661±0.129a	8.757±1.048b	77.583±0.950b

Mean ± SD is calculated from duplicate measurements.

Values with different letters in the same column are significantly different (*p*< 0.05).

## Conclusion

This work highlighted changes of crystalline structure and physicochemical properties of starches extracted from *Pueraria lobata* var. *thomsonii* under water deficit. Under water deficit, there was an increase in small starch granules and a decrease in large starch granules. Additionally, there was a decrease in total starch content and amylose content, while transparency of amylopectin increased. Water deficit resulted in the crystal type of *P*. *thomsonii* starch granules changed from C_B_-type to C_A_-type. The transformation of the crystal type induced changes in the DSC heat flow curve, resulting in a shift from a single peak to a double peak. Additionally, the behavior of starch gelatinization is altered, and there is an increase in the content of resistant starch. Water deficit can regulate starch synthesis and crystal assembly in organisms, thereby affecting the functional properties of starch.
